# Exercise-based cardiac rehabilitation for patients with atrial fibrillation: a narrative review

**DOI:** 10.1093/ehjopen/oeaf025

**Published:** 2025-03-14

**Authors:** Benjamin J R Buckley, Liz van Hout, Charlotte Fitzhugh, Gregory Y H Lip, Rod S Taylor, Dick H J Thijssen

**Affiliations:** Liverpool Centre for Cardiovascular Science, University of Liverpool, Liverpool John Moores University and Liverpool Heart & Chest Hospital, Liverpool, UK; Liverpool John Moores University, Research Institute for Sport and Exercise Sciences, Byrom Street, Liverpool L3 3AF, UK; Department of Medical BioSciences, Cardiovascular Physiology, Radboud University Medical Center, Geert Grooteplein Zuid 10, 6525 GA Nijmegen, The Netherlands; Liverpool John Moores University, Research Institute for Sport and Exercise Sciences, Byrom Street, Liverpool L3 3AF, UK; Liverpool Centre for Cardiovascular Science, University of Liverpool, Liverpool John Moores University and Liverpool Heart & Chest Hospital, Liverpool, UK; Danish Center for Health Services Research, Department of Clinical Medicine, Aalborg University, Selma Lagerløfs Vej 249, 9260 Gistrup, Aalborg, Denmark; MRC/CSO Social and Public Health Sciences Unit & Robertson Centre for Biostatistics, School of Health and Well Being, University of Glasgow, Glasgow, UK; Liverpool Centre for Cardiovascular Science, University of Liverpool, Liverpool John Moores University and Liverpool Heart & Chest Hospital, Liverpool, UK; Department of Medical BioSciences, Cardiovascular Physiology, Radboud University Medical Center, Geert Grooteplein Zuid 10, 6525 GA Nijmegen, The Netherlands

**Keywords:** Atrial fibrillation, Exercise, Physical activity, Pathophysiology

## Abstract

The role of physical activity (i.e. any bodily movement that requires energy expenditure) and exercise (i.e. planned, structured, and repetitive physical activity to improve/maintain fitness) in the primary and secondary prevention of atrial fibrillation (AF) is increasingly recognized. Physical activity has been associated with lower risks to develop AF and associated complications (e.g. stroke, heart failure, and myocardial infarction). Exercise-based cardiac rehabilitation (ExCR) is increasingly examined in the treatment of AF and sometimes combined with rhythm control strategies (e.g. catheter ablation). Nonetheless, several important clinical, practical, and mechanistic questions remain not fully understood. This state-of-the-art review first provides a contemporary update on the evidence base for the clinical effects of ExCR in AF. Despite the ongoing need for high-quality studies, existing randomized controlled trials and cohort studies suggest ExCR reduces AF burden, lowers risks for major adverse cardiovascular events, and improves health-related quality of life. Second, to facilitate implementation of ExCR, we have observed comparable effects of distinct exercise protocols (e.g. type of training and centre-/home-based) and discussed similarity of effectiveness across patient characteristics (e.g. age, sex, and AF subtype). Critically, we have discussed potential barriers that may prohibit the uptake of ExCR for patients with AF, categorized at clinician- (e.g. referral and training), patient- (e.g. motivation, transportation, and psychosocial factors), and system-levels (e.g. insurance and resources). Third, we have summarized the potential mechanisms underlying these effects of ExCR, classified by their potential role in reducing AF burden (e.g. atrial/ventricular function, autonomic balance, and inflammation) and lowering risks for adverse events (e.g. modifiable risk factors, vascular function, and thrombogenesis). Based on the increasing evidence for clinical benefits, e.g. improved health-related quality of life and better clinical outcomes, we advocate stronger focus on regular physical activity and referral to multidisciplinary ExCR for sustainable lifestyle changes within the ESC AF-CARE pathway for the prevention and treatment of AF.

## Graphical Abstract

**Graphical Abstract:**
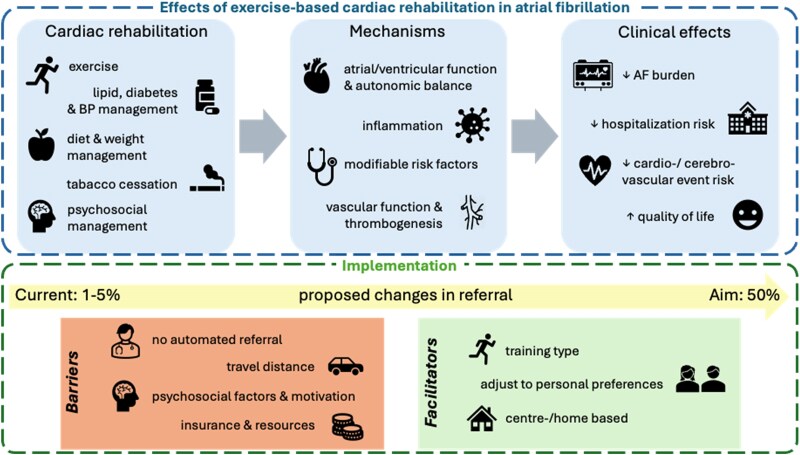

## What is the global burden of disease for AF?

Atrial fibrillation (AF) is the most common cardiac arrhythmia, with prevalence expected to rise exponentially within an ageing population.^1^ The presence of AF is associated with increased risk for cardio-/cerebrovascular mortality and morbidity^2^ and developing dementia,^3^ but also associated with presence of anxiety and depression. Consequently, these health effects contribute to an impaired quality of life for patients with AF.

The management of AF has progressed to a more holistic or integrated care approach. The 2024 ESC guidelines propose the AF-CARE pathway (an acronym revision of the evidence-based ABC pathway^4^): [C] Comorbidity and risk factor management, [A] Avoid stroke and thromboembolism, [R] Reduce symptoms by rate and rhythm control, [E] Evaluation and dynamic reassessment.^5^ The 2023 ACC/AHA/HRS/ACCP guidelines promote such holistic care as ‘SOS’.^6^

Adherence to such multidisciplinary approaches has been associated with improved clinical outcomes,^7,8^ leading to its recommendations in international guidelines. Nonetheless, residual risk of complications in patients with AF remains present, in addition to potential drug side effects, impact of comorbidities, relatively high post-ablation AF-recurrence rates, and high healthcare costs.^9^

An increasing number of studies explore the role of physical activity (PA; i.e. any bodily movement that increases energy expenditure) and exercise (i.e. planned, structured, and repetitive physical activity to increase or maintain fitness). Regular PA is well established to enhance cardiovascular health^10^ and lower cardiovascular risk,^11^ and is associated with a lower risk for AF.^12–14^ Exercise-based cardiac rehabilitation (ExCR) is a multicomponent intervention that includes exercise training and PA promotion, but also health education, cardiovascular risk management, and psychological support. International guidelines in cardiology,^15^ including the recent ESC guidelines for management of AF,^5^ consistently give ExCR their highest recommendation (Class 1: treatment should be recommended) in the treatment of patients with myocardial infarction or following coronary interventions and heart failure.

The potential role of ExCR in AF has long been ignored, as supported by the remarkably low uptake of ExCR in patients with AF (0–5%).^16^ In the past decade, an increasing number of studies have demonstrated potential benefits of ExCR,^17^ which aligns within the current holistic AF-CARE pathway approach in treating AF. Although our understanding of ExCR in patients with AF has improved, many questions remain unanswered.

In this narrative review, we first provide a contemporary update on the evidence base for the clinical effects of PA and ExCR in AF. Second, to facilitate implementation of ExCR in AF, we will discuss optimization of exercise protocols, highlight between-group differences, and discuss potential barriers. Finally, we will summarize the mechanisms underlying these clinical effects, and provide recommendation for future work.

### Methodology

Although we have not adopted a pre-registered systematic review process, this narrative review was informed by prior systematic reviews. This review therefore included trials from a recent Cochrane systematic review of ExCR for adults with AF,^18^ as well as two other, non-Cochrane systematic reviews investigating exercise-based CR or physical activity^19^ and different types of exercise interventions^20^ for people with AF. Building on the methodology outlined in these reviews, we included both original trials from the Cochrane review and additional prospective and retrospective studies published that examined the impact of exercise or physical activity on health outcomes for people with AF. Second, we have used the following search strings ‘physical activity OR exercise OR sport*’ and ‘exercise-based cardiac rehabilitation OR cardiac rehab* OR rehab*’ to ensure any other contemporary studies were considered.

## Role for physical activity and exercise in AF

### Is physical activity related to the primary prevention of AF?

Emerging evidence linked regular PA engagement to the primary prevention of AF, with even light-to-moderate-intensity PA being associated with lower AF incidence.^12^ A meta-analysis including 22 case–control and observational studies (*n* = 656 750)^21^ revealed that an ‘inactive lifestyle’ was associated with a 2.5-fold increased lifetime risk of developing AF, a risk that was independent of sex. In a recent UK Biobank cohort analysis (*n* = 402 406), Elliott *et al*.^13^ found that achieving >1500 MET-min/week (metabolic equivalents) of activity was associated with a 15% lower risk of incident AF vs. no activity. Lower risks for incident AF in relation to regular PA have also been observed in those with disease (e.g. type 2 diabetes).^22^

### How is the spectrum of physical activity related to AF development?

**Figure 1:**
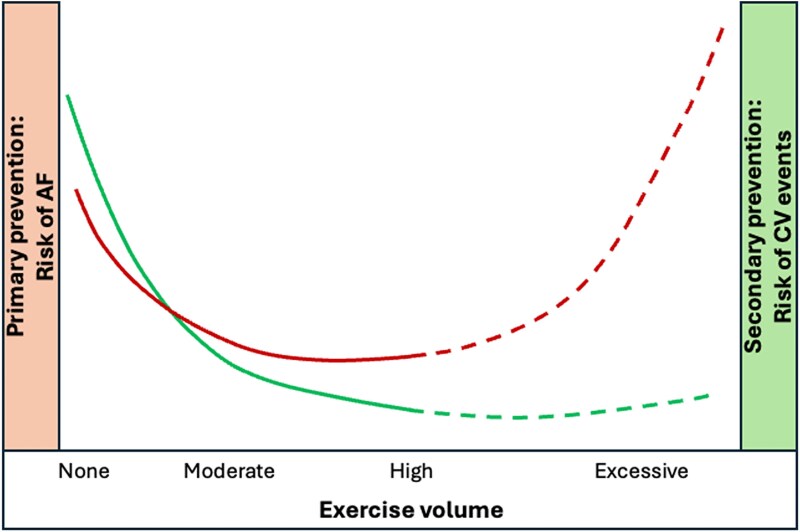
Presentation of the dose–response relationship between the volume of exercise and the primary [red line (line starting at the lower point on the left): risk of AF in general population] and secondary prevention [green line (line starting at the higher point on the left): risk of AF recurrence and CV events] in relation to atrial fibrillation. Moderate and high exercise volumes reflect meeting 1–2 or 3–4 times the WHO guidelines on physical activity, respectively. The dashed line represents the hypothetical part on the risks associated with excessive volumes of exercise, for which relatively few studies have provided insight.

The observation that regular PA is protective for incident AF also raises questions regarding its dose–response relation. Below, we have addressed this topic by discussing the potential negative effects of sedentary behaviour and excessive endurance training, but also the impact of sex differences (Section 2.2). Despite the potentially complex nature of the dose–response relationship between PA, exercise, and AF, the majority of studies suggest beneficial effects in the primary prevention of AF when adopting or modestly exceeding (2–3 times) current guideline levels (*Figure 1*).

#### Sedentary behaviour

Although high volumes of sedentary behaviour (e.g. activities at low energy expenditure in a sitting or lying position) increase risks of cardiovascular mortality and morbidity,^23^ few have examined this in the context of AF. A previous study in 14 458 adults reported that greater frequency of TV watching was associated with an increased risk of AF.^24^ However, TV watching is a poor proxy for sedentary time. A more recent study, using accelerometry in older women, found that the most sedentary quartile (11 h/day) showed a HR of 2.19 for developing AF compared to the least sedentary quartile (7.3 h/day), but this effect disappeared when controlling for PA.^25^

#### Extreme volumes of exercise

With the growing popularity of endurance exercise, there is a lively debate whether high volumes of exercise may be detrimental to cardiovascular health.^26^ Regarding AF, there seems consensus that high volumes of endurance exercise are associated with a counterintuitive increased risk for AF.^27^ A previous meta-analysis and meta-regression^28^ revealed 2.46 increased odds of AF in athletes compared to non-athlete controls. Whilst meta-regression suggested those participating in mixed sports demonstrated the highest risk for AF, others suggest that specifically endurance exercise is associated with the highest risk.^29^ In addition, Morseth *et al*.^30^ found that participating in vigorous-intensity PA diminishes beneficial effects of regular walking/cycling on risk of incident AF. A lack of prospective study of objective exercise volume and AF occurrence makes it difficult to truly understand and identify factors interacting with this increased risk. Nonetheless, an important observation is that sex may interact with the risk of AF in relation to high levels of physical activity. Based on self-reported physical activity levels in 402 406 individuals from the UK Biobank cohort, Elliott *et al*.^13^ reported that excessive volumes of vigorous physical activity are associated with incident AF in men, but not in women. Future studies are warranted to better understand these observations, including understand the potential underlying reasons. Taken together, despite these observations of an increased risk of AF with excessive exercise, these volumes far exceed existing guidelines and it appears to take years of training for such elevated risk.

### What is the effect of exercise-based cardiac rehabilitation in AF?

#### Randomized clinical trials

Although being a core component, ExCR goes beyond exercise training alone and also targets other lifestyle factors, including smoking, overweight/obesity, and excessive alcohol intake. A recent Cochrane systematic review and meta-analysis with meta-regression included 20 randomized clinical trials (RCTs) investigating the effects of ExCR compared to exercise control for participants with AF (*n* = 2039).^18^ There was high heterogeneity in trial intervention length (8–52 weeks), frequency (1–7 sessions/week), and length (15–90 min/session). Exercise type consisted of aerobic exercise at either light-, moderate-, or high-intensity, a combination of aerobic- and resistance-based training. Four studies investigated non-traditional exercise programmes, including Qi-gong, inspiratory muscle training, and yoga.

Meta-analysis showed little to no difference in all-cause mortality between ExCR and control groups [relative risk (RR) 1.06, 95% confidence interval (CI) 0.76–1.49] and serious adverse events (including any untoward medical occurrence that was life-threatening, resulting in death, or that was persistent or leading to significant disability or hospitalization) (RR 1.30, 95% CI 0.63–2.67). However, the certainty evidence of these outcomes was rated low due to limited number of events and trials contributing to the effect estimate. An important caveat is the limited follow-up duration within most included trials, as clinical outcomes may occur later in life. We therefore strongly recommend future studies to include a sufficiently long follow-up period to understand the potential of ExCR on adverse clinical endpoints.

ExCR was associated with reduced AF recurrence (RR 0.70, 95% CI 0.56–0.88) and patient-reported AF symptom severity and burden. This supports prior epidemiological work suggesting a beneficial role of exercise in managing AF.^31^ To extend this observation, the Cochrane review showed improvements in health-related quality of life for those undergoing ExCR in the mental health subscale, but not the physical. Nonetheless, measures of exercise capacity (e.g. peak oxygen uptake and 6 min walk test) showed statistically significant (and clinically meaningful) improvements following ExCR.

One trial included in the Cochrane review was performed by Elliott *et al*.^32^ who randomized 120 patients with symptomatic paroxysmal or persistent AF to a tailored exercise and physical activity intervention or control group (i.e. educational sessions about physical activity). This trial showed improvements in exercise capacity and quality of life (QoL) following ExCR, but also a significant reduction in objectively measured AF recurrence. In support of these clinical effects of ExCR, Malmo *et al*.^33^ focused on the short-term effects of high-intensity aerobic interval training and demonstrated a significant reduction in AF episodes. A more recent RCT compared high-intensity interval training vs. moderate-intensity continuous training (MICT) in 86 participants with AF. Despite a substantially lower total exercise volume, the authors found that high-intensity interval training was as efficacious as moderate-intensity training in improving functional capacity, quality of life, resting heart rate, and physical activity levels.^34^ Another study by Luo *et al*.^35^ examined the long-term effects of ExCR on outcome of cardiovascular death and heart failure hospitalization. They highlighted the potential of ExCR in reducing severe cardiovascular events, although the difference in mortality was not statistically significant and underpowered.

#### Cohorts

In a cohort study conducted by Pathak *et al*.,^36,37^ it was found that greater cardiorespiratory fitness (CRF) was associated with increased freedom of AF and for every 1 MET increase in CRF (via exercise training), AF recurrence was reduced by 9%. Similarly, in >64 500 adults, Qureshi *et al*.^38^ observed that every 1 MET increase in CRF was associated with a 7% lower risk of incident AF. More recently, Garnvik *et al*.^39^ collected self-reported PA and estimated CRF in 1117 patients with prevalent AF over ∼8 years. Primary findings showed that meeting the guideline 150 min/week of moderate-intensity PA resulted in a 45% and 50% lower risk of all-cause and cardiovascular disease mortality, respectively, in comparison to those who were inactive. In addition, each 1 MET increase in CRF was associated with 12% lower all-cause mortality and 15% lower cardiovascular disease mortality. Even achieving less than the guideline PA levels associated with a lower risk of mortality compared with inactive patients, advocating that, even below the recommended levels of PA, some PA is better than nothing for secondary prevention of AF.

In a large cohort study including >66 000 patients with a recent diagnosis of AF, it was found that those who initiated exercise following their diagnosis, or maintained MICT, had lower rates of heart failure, stroke, and mortality.^40^ Similar observations were found in the HUNT3 study^39^ where it was observed that patients with AF who engaged in MICT had lower long-term risks of all-cause mortality and cardiovascular disease in comparison to the non-exercise group. Finally, recent epidemiological studies have shown that ExCR associates with significantly lower risk of clinical events and morbidity,^41^ as well as lower risk for AF progression vs. matched controls.^31^

Collectively, cohort studies and RCTs investigating ExCR for patients with AF support its use as a valuable component of comprehensive AF management.^42^ Beneficial effects have been reported on AF recurrence, exercise capacity, and QoL, though the impact on clinical events (e.g. mortality and serious adverse events) remains uncertain. The evidence base, whilst promising, requires further high-quality RCTs with standardized protocols and longer follow-up periods to confirm the role of ExCR in AF treatment paradigms, and may identify exercise modalities and intensities that yield the most significant benefits for AF patients and any difference in subgroups to aid personalization and widen options for patients.

## Implications for clinical guidance and implementation

### Are benefits consistent across patient and intervention characteristics?

#### Patient characteristics

Whilst individual RCTs are underpowered to assess the impact of patient characteristics, the Cochrane systematic review of ExCR for AF (*n* = 2039) showed the benefits of ExCR were consistent across different patient characteristics including age, sex, AF subtype (paroxysmal/persistent/permanent), and catheter ablation treatment. Sufficiently powered RCTs and/or individual patient data meta-analytical approach may assist in better understanding the role of patient characteristics.

#### Exercise characteristics

Based on frequently studied cardiovascular conditions, meta-regression indicates that total energy expenditure of an exercise programme is the strongest predictor for an increase in exercise capacity.^43^ Methodological limitations of existing studies (e.g. sample size, lack of follow-up, and limited variation in exercise volumes) prohibit a valid evaluation of this in AF. Over the years, high-intensity interval training has received substantial attention as a time-efficient and potentially effective strategy in cardiac rehabilitation (see review).^44^ Studies examining the impact of high-intensity interval training (∼1 h/week) in patients with AF have revealed its potential to reduce AF burden.^33^ However, both high-intensity interval and MICT seem equally effective to improve fitness and disease-specific outcomes.^34^ Taken together, there is currently no strong evidence that one type, mode, and/or intensity of exercise is superior (at group level) in patients with AF.

#### Home- vs. centre-based

Due to recent advances in technology, partly accelerated by the SARS-CoV-2 pandemic, remote digital support is an expanding approach. A large systematic review with meta-analysis (96 studies) concluded that telemonitoring was an effective intervention, reducing mortality and improving self-management of the disease, with patients reporting good satisfaction and adherence.^45^ A recent Cochrane review (24 trials; *n* = 3046) found comparable benefits of home- vs. centre-based ExCR in all-cause mortality (12 months follow-up) and health-related quality of life (24 months follow-up).^46^ Whilst it seems crucial to consider the risks and pitfalls of these wearables, these observations together support the ongoing use of digital health (e.g. wearables) to support remote delivery in treatment of AF.

### What are current barriers preventing wider implementation?

**Figure 2:**
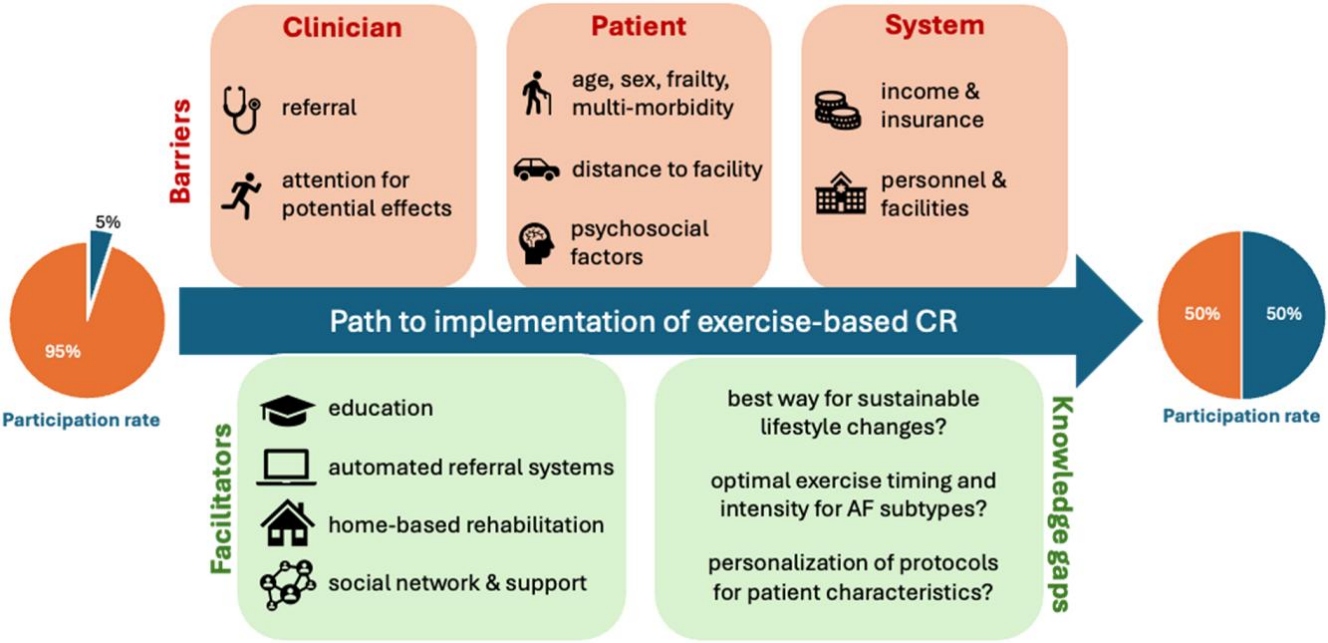
Presentation of barriers (top) and facilitators + future direction (bottom) for the wider implementation and referral to ExCR in patients with AF. Barriers for implementation have been categorized into factors related to the clinician, patient, and system. The current and proposed participation rate target has been presented in the middle.

Despite the strong evidence supporting benefits of ExCR and international clinical guidelines recommending ExCR participation, uptake of ExCR in eligible patients is low.^16^ Whilst barriers are likely interrelated, studies have categorized barriers of ExCR uptake according to clinician-, patient-, and health system-level factors (*Figure 2*). Although evidence on ExCR uptake in AF is scarce, and uptake in AF is amongst the lowest of cardiovascular diseases (<1–5%),^16^ it appears that barriers are comparable to those seen in coronary heart disease (CHD) and heart failure (HF) patients.^47^

#### Healthcare professional-level

One potential barrier relates to the relatively few studies examining, and subsequently supporting, the effects of ExCR in patients with AF. This specifically relates to sufficiently powered RCTs with long-term follow-up to establish the clinical benefits of ExCR, and identify strategies to optimize the timing and content of the exercise protocol for AF. Indeed, prescription to ExCR is not specifically mentioned in ABC-pathway or AF-CARE model.^5^ Furthermore, presence of AF is associated with lower referral rates to ExCR in those with coronary artery disease. This highlights the need to improve education about the effects of PA in AF, during both general medical and cardiology training. Another potential solution relates to adopting an automated referral system combined with a patient discussion, as such approaches lead to superior referral rates (review),^48^ with increases up to 45%.^49^

#### Patient-level

Multiple patient-level factors have been identified, with non-referral being more common with older age, female, frailty, and multi-morbidity.^16^ Interestingly, studies in frail patients with heart failure revealed cardiac rehabilitation to be effective, if not superior compared to non-frail participants.^50^ This suggests that benefits of ExCR are robust and seem present across (sub)groups. The distance to the rehabilitation facility is another frequently reported patient-level barrier,^16^ for which remote home-based rehabilitation seems a feasible and potentially successful facilitator. Other relevant patient-level barriers relate to time restrictions and lack of motivation. Pertaining to these barriers, we reported that clinical benefits seem equally present across exercise protocols, but also between centre- vs. home-based rehabilitation (Section 2.1). This allows to acknowledge patients’ personal barriers and/or preferences.

A final patient-related barrier relates to psychosocial factors, including caregiver support, socioeconomic status, and self-efficacy. Indeed, a recent Cochrane review reported potential for positive effects for social network and social support interventions, but also highlighted the lack of sufficient evidence for conclusive support.^51^

#### System-level

Previous work indicated that insufficient personnel and facilities represent an important system-level barrier to prescribe ExCR in low- and middle-income countries (review),^52^ whilst high-income countries also experience this barrier. Another system-level factor is the variation in insurance systems, which unfavourably affects lower socioeconomic groups. To highlight its complexity, even in countries where ExCR is financially covered, lower income is associated with lower referral rates.^16^

## Mechanisms explaining the benefits of exercise for AF

### What mechanisms are likely related to AF burden or recurrence?

#### Atrial remodelling

Pathological changes in atrial structure (e.g. enlarged and fibrosis) and function (e.g. lower reservoir and strain) are associated with development and progression of AF, whilst atrial function seems strongly related to exercise intolerance.^53^ Whilst left ventricular adaptation is widely examined, only few studies explored the effects of exercise training on atrial health (review).^54^ Regarding atrial fibrosis, i.e. a central proponent of the atrial arrhythmogenic phenotype, endurance training unlikely reverses this process.^55^ Alternatively, weight loss and exercise training may promote reversal of atrial enlargement in patients with AF.^56^ Furthermore, exercise training has also been linked to improved left atrial function.^33^ However, despite the reductions in AF burden, not all training studies in AF report atrial adaptations.^32^ These findings suggest that exercise training in patients with AF can contribute to atrial remodelling, but this is not a prerequisite for clinical benefits.^54^

#### Autonomic imbalance

The activity of the autonomic nervous system plays a crucial role in the pathogenesis of AF. Several meta-analyses have evaluated the effects of exercise training in various populations with cardiovascular disease or risk on autonomic balance, with heart rate variability a popular outcome. Whilst these meta-analyses emphasize a lack of robust clinical studies, most report the ability of exercise training to improve autonomic balance.^57^ In patients with AF, exercise training seems to lower resting heart rate^58^ and increase heart rate variability, the latter indicative for increased vagal tone.

#### Inflammation

In line with other cardiovascular diseases, presence of inflammation is a well-established risk factor for the development of AF. The effects of exercise on inflammatory markers are complex with conflicting results. This may, at least in part, be related to the duration (acute vs. chronic), intensity, and volume of exercise.^59^ Regular PA or exercise, not performed at excessive intensity/volume, seems associated with the suppression of pro-inflammatory cytokine production, enhancing anti-inflammatory mediators, antioxidant development, and promoting fibrinolytic activity.^60^

### What mechanisms are likely related to clinical events?

#### Traditional cardiovascular risk factors

The pathogenesis of arrhythmia and development of clinical events is strongly related to traditional cardiovascular risk factors. This includes hypertension, dyslipidaemia, obesity, smoking, low fitness, and diabetes mellitus (review).^61^ Engagement in regular PA is demonstrated to improve risk factors, which, at least partly explains the benefits of PA in protecting against cardiovascular events. To reinforce these observations in AF, Pathak *et al*.^37^ examined the effects of a structured exercise programme in 308 participants with AF, and found improvements in traditional cardiovascular risk factors. Moreover, they also demonstrated significantly larger effect sizes in those who improved their fitness the most^37^; suggesting that clinical benefits from exercise training relate to improvements in fitness and risk factors.

#### Endothelial function

Through their direct effects on the endothelium, risk factors and inflammation may contribute to developing AF (and clinical events) through endothelial dysfunction. Indeed, patients with AF demonstrate an impaired endothelial function,^62^ whilst measures of vascular health independently predict AF. Regular PA is well established to improve vascular function and structure, most likely through the repeated exposure to haemodynamic stimuli (e.g. shear stress).^10^ Supporting this hypothesis, a recent RCT (*n* = 74) evaluated the impact of 6–12-month exercise training in patients with AF and found improvements in blood biomarkers of endothelial function.^63^

#### Thrombogenesis

The prothrombotic state in AF, strongly related to development of stroke and thromboembolism, occurs as a consequence of multifaceted interactions known as Virchow’s triad of hypercoagulability, arterial abnormalities, and intra-atrial stasis. In addition to the impact on arterial abnormalities (see ‘endothelial dysfunction’), PA and exercise in patients with cardiovascular disease,^63^ including AF,^64^ are able to affect the other components of Virchow’s triad, e.g. platelet function and thrombogenic factors.^63,64^

**Figure 3:**
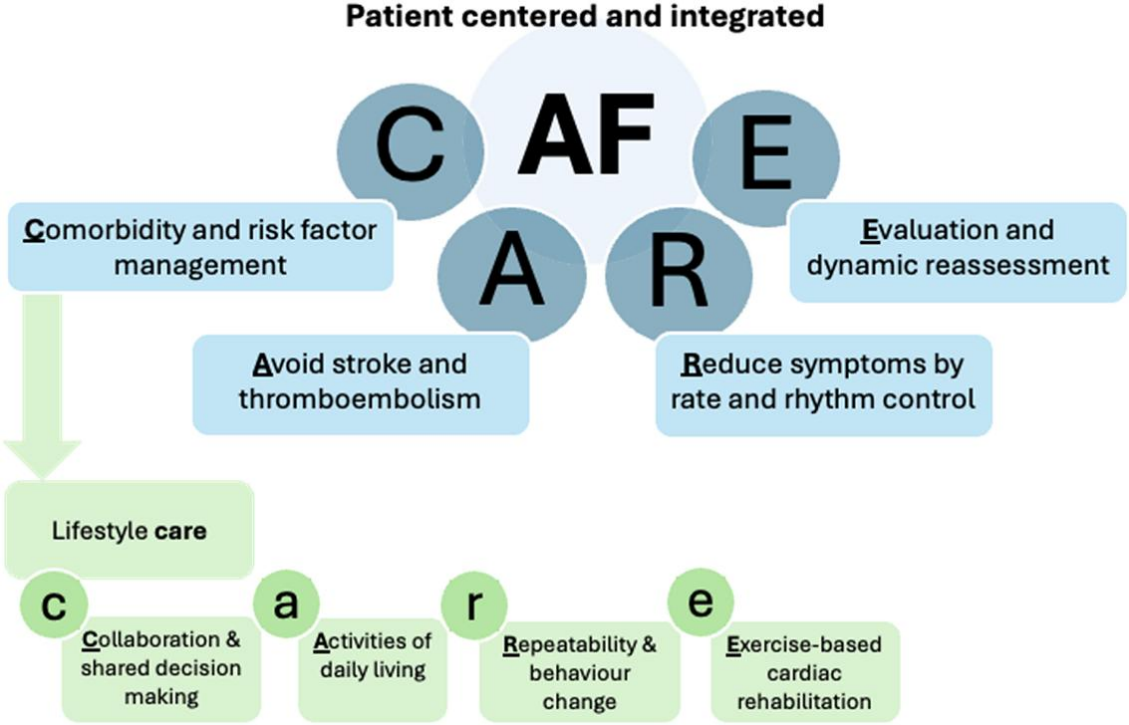
Presentation of the holistic AF-CARE pathway in the treatment of AF, with expansion of the comorbidity and risk factor management (‘C’), with specific focus on the promotion of an active lifestyle. Specifically, we have highlighted four key pillars to promote an active lifestyle following the ‘lifestyle-care’ pathway; [c] collaboration for shared-decision making, [a] activities in daily living, [r] repeatability in engaging in this lifestyle for sustainable changes, and [e] exercise-based cardiac rehabilitation.

Taken together, potential mechanisms underlying the effects of regular exercise and physical activity primarily relate to (i) reducing AF burden or recurrence, or (ii) lowering risks for clinical events (*Figure 3*). Nonetheless, some mechanisms likely show interaction effects and, therefore, contribute to both endpoints (e.g. inflammation and risk factors). Future prospective studies are warranted to truly understand these underlying mechanisms in AF.

## Future directions

The evidence supporting benefits of ExCR for people AF is increasingly promising and includes reduction in AF burden and cardiovascular risk, and improvement in health-related quality of life. Nonetheless, definitive evidence is needed through well conducted multicentre RCTs adequately powered to evaluate relevant clinical outcomes. Future trials should also address the following questions: optimal timing of ExCR prescription (e.g. ablation), cost-effectiveness of ExCR, and AF populations who benefit from ExCR (e.g. AF subtype, sex, and frailty) (*Table 1*). Furthermore, at the patient-level, we anticipate that current advances in digital health for home-based treatment alleviate several barriers (e.g. transportation, time constraints, and individual supervision). Moreover, the robust effects across various exercise protocols allow for personalized treatment programmes (e.g. patient preferences and facilities). We recommend multiple strategies, rather than a single ‘silver bullet’, for successful clinical implementation.

**Table 1:** Summary of findings

Key findings
Regular physical activity (PA) lowers the risk of developing AF, with even light-to-moderate-intensity PA showing protective effects.^13^
Exercise-based cardiac rehabilitation (ExCR) reduces AF burden and AF severity, improves health-related quality of life, and enhances exercise capacity.^18^
Both high-intensity interval training (HIIT) and moderate-intensity continuous training (MICT) appear effective for AF patients.^34^
Home-based ExCR appears as effective as centre-based programmes, supporting remote delivery for CVD patients in general.^46^
Barriers to ExCR uptake exist at clinician, patient, and system-levels, contributing to low referral and adherence rates for patients with CVD in general (*Figure 2*). ExCR is not yet indicated for patients with AF, yet tailored exercise is given a Class-I recommendation in the latest ESC guidelines.^65^
Gaps for future research
Need for large, multicentre RCTs with long-term follow-up to confirm clinical event reduction from ExCR, as suggested in cohort data.^19,41^
Optimal timing and intensity of ExCR prescription for different AF subtypes remain unclear.
Cost-effectiveness and economic burden of implementing ExCR in AF patients require further study and whether certain subgroups should be prioritized.
Personalization of exercise protocols based on patient characteristics (e.g. sex, frailty, and AF subtype) is not well understood.
Effectiveness of mobile health interventions and remote monitoring for ExCR implementation needs further validation in patients with AF.
Role of ExCR in combination with pharmacological and rhythm control strategies (e.g. ablation) remains to be fully explored.
Longitudinal studies needed to understand dose–response relationships of PA in AF patients.

There are also substantial challenges in implementation of ExCR in current clinical treatment of AF. Acknowledging the complexity and interaction of the various barriers, we highlight the potential of automated referral combined with patient instruction. This may require integration of ExCR in treatment guidelines. Despite the undisputed benefits of the integrative multidisciplinary pathway in treating AF, relatively little attention is present on *promoting* lifestyle changes and strategies to achieve these. Therefore, aligned with C (comorbidity and risk factor management) being listed as the first element of the AF-CARE model (*Figure 3*),^5^ we propose clinicians to more strongly focus on the promotion of physical activity and exercise for people with AF. This is particularly timely with the recent Cochrane findings and 2024 ESC guidelines giving a Class-I recommendation for tailored exercise programmes. Specifically, we have highlighted four key pillars that seem crucial to promote an active lifestyle following the ‘lifestyle-care’ pathway; [c] collaboration for shared-decision making, [a] activities in daily living, [r] repeatability in engaging in this lifestyle for sustainable changes, and [e] exercise-based cardiac rehabilitation (*Figure 3*).

In conclusion, based on the increasing evidence for PA and exercise leading to clinical benefits, we advocate for future research and consideration of ExCR within the multidisciplinary holistic or integrated care management pathways to enhance its implementation.

## Data Availability

No new data were generated or analysed in support of this research.

## Lead author biography

Dr Benjamin J.R. Buckley is a Senior Lecturer at the Liverpool Centre for Cardiovascular Science and a Fellow of the European Society of Cardiology. His research focuses on preventive cardiology, with expertise in cardiovascular epidemiology. He is particularly interested in the role of lifestyle in preventing and managing chronic conditions, as well as brain-heart interactions. Beyond academia, he enjoys spending time in nature, particularly trail running and climbing.

## References

[1] Linz D, Gawalko M, Betz K, Hendriks JM, Lip GYH, Vinter N, Guo Y, Johnsen S. Atrial fibrillation: epidemiology, screening and digital health. Lancet Reg Health Eur 2024;37:100786. 38362546 10.1016/j.lanepe.2023.100786 PMC10866942

[2] Odutayo A, Wong CX, Hsiao AJ, Hopewell S, Altman DG, Emdin CA. Atrial fibrillation and risks of cardiovascular disease, renal disease, and death: systematic review and meta-analysis. BMJ (Clinical research ed 2016;354:i4482. 10.1136/bmj.i4482 27599725

[3] Singh-Manoux A, Fayosse A, Sabia S, Canonico M, Bobak M, Elbaz A, Kivimäki M, Dugravot A. Atrial fibrillation as a risk factor for cognitive decline and dementia. Eur Heart J 2017;38:2612–2618. 28460139 10.1093/eurheartj/ehx208 PMC5837240

[4] Potpara T, Romiti GF, Sohns C. The 2024 European Society of Cardiology guidelines for diagnosis and management of atrial fibrillation: a viewpoint from a practicing clinician’s perspective. Thromb Haemost 2024;124:1087–1094. 39374908 10.1055/a-2434-9244

[5] Van Gelder IC, Rienstra M, Bunting KV, Casado-Arroyo R, Caso V, Crijns HJGM, De Potter TJR, Dwight J, Guasti L, Hanke T, Jaarsma T, Lettino M, Løchen M-L, Lumbers RT, Maesen B, Mølgaard I, Rosano GMC, Sanders P, Schnabel RB, Suwalski P, Svennberg E, Tamargo J, Tica O, Traykov V, Tzeis S, Kotecha D; ESC Scientific Document Group. 2024 ESC Guidelines for the management of atrial fibrillation developed in collaboration with the European Association for Cardio-Thoracic Surgery (EACTS). Eur Heart J 2024;45:3314–3414. 39210723 10.1093/eurheartj/ehae176

[6] Joglar JA, Chung MK, Armbruster AL, Benjamin EJ, Chyou JY, Cronin EM, Deswal A, Eckhardt LL, Goldberger ZD, Gopinathannair R, Gorenek B, Hess PL, Hlatky H, Hogan G, Ibeh C, Indik JH, Kido K, Kusumoto F, Link MS, Linta KT, Marcus GM, McCarthy PM, Patel N, Patton KK, Perez MV, Piccini JP, Russo AM, Sanders P, Streur MM, Thomas KL, Times S, Tisdale JE, Valente AM, Van Wagoner DR; Peer Review Committee Members. 2023 ACC/AHA/ACCP/HRS guideline for the diagnosis and management of atrial fibrillation: a report of the American College of Cardiology/American Heart Association Joint Committee on Clinical Practice Guidelines. Circulation 2024;149:e1–e156. 38033089 10.1161/CIR.0000000000001193 PMC11095842

[7] Treewaree S, Lip GYH, Krittayaphong R. Non-vitamin K antagonist oral anticoagulant, warfarin, and ABC pathway adherence on hierarchical outcomes: win ratio analysis of the COOL-AF registry. Thromb Haemost 2024;124:69–79. 37625457 10.1055/s-0043-1772773

[8] Romiti GF, Pastori D, Rivera-Caravaca JM, Yew Ding W, Gue YX, Menichelli D, Gumprecht J, Kozieł M, Yang P-S, Guo Y, Lip GYH, Proietti M. Adherence to the ‘atrial fibrillation better care’ pathway in patients with atrial fibrillation: impact on clinical outcomes—a systematic review and meta-analysis of 285,000 patients. Thromb Haemost 2022;122:406–414. 34020488 10.1055/a-1515-9630

[9] Ding WY, Lane DA, Gupta D, Huisman MV, Lip GYH, Investigators G-A. Incidence and risk factors for residual adverse events despite anticoagulation in atrial fibrillation: results from phase II/III of the GLORIA-AF registry. J Am Heart Assoc 2022;11:e026410. 35876418 10.1161/JAHA.122.026410 PMC9375480

[10] Green DJ, Hopman MT, Padilla J, Laughlin MH, Thijssen DH. Vascular adaptation to exercise in humans: role of hemodynamic stimuli. Physiol Rev 2017;97:495–528. 28151424 10.1152/physrev.00014.2016 PMC5539408

[11] Piercy KL, Troiano RP, Ballard RM, Carlson SA, Fulton JE, Galuska DA, George SM, Olson RD. The physical activity guidelines for Americans. JAMA 2018;320:2020–2028. 30418471 10.1001/jama.2018.14854 PMC9582631

[12] Mozaffarian D, Furberg CD, Psaty BM, Siscovick D. Physical activity and incidence of atrial fibrillation in older adults: the cardiovascular health study. Circulation 2008;118:800–807. 18678768 10.1161/CIRCULATIONAHA.108.785626 PMC3133958

[13] Elliott AD, Linz D, Mishima R, Kadhim K, Gallagher C, Middeldorp ME, Verdicchio CV, Hendriks JML, Lau DH, Gerche AL, Sanders P. Association between physical activity and risk of incident arrhythmias in 402 406 individuals: evidence from the UK Biobank cohort. Eur Heart J 2020;41:1479–1486. 31951255 10.1093/eurheartj/ehz897

[14] Ahn HJ, Choi EK, Rhee TM, Choi J, Lee K-Y, Kwon S, Lee S-R, Oh S, Lip GYH. Accelerometer-derived physical activity and the risk of death, heart failure, and stroke in patients with atrial fibrillation: a prospective study from UK Biobank. Br J Sports Med 2024;58:427–434. 38418213 10.1136/bjsports-2023-106862

[15] Taylor RS, Dalal HM, McDonagh STJ. The role of cardiac rehabilitation in improving cardiovascular outcomes. Nat Rev Cardiol 2022;19:180–194. 34531576 10.1038/s41569-021-00611-7 PMC8445013

[16] Eijsvogels TMH, Maessen MFH, Bakker EA, Meindersma EP, van Gorp N, Pijnenburg N, Thompson PD, Hopman MTE. Association of cardiac rehabilitation with all-cause mortality among patients with cardiovascular disease in The Netherlands. JAMA Netw Open 2020;3:e2011686. 32716516 10.1001/jamanetworkopen.2020.11686 PMC12124693

[17] Vermeer JR, van den Broek J, Dekker LRC. Impact of lifestyle risk factors on atrial fibrillation: mechanisms and prevention approaches—a narrative review. Int J Cardiol Cardiovasc Risk Prev 2024;23:200344. 39534719 10.1016/j.ijcrp.2024.200344 PMC11555354

[18] Risom SS, Zwisler A-D, Johansen PP, Sibilitz KL, Lindschou J, Gluud C, Taylor RS, Svendsen JH, Berg SK. Exercise-based cardiac rehabilitation for adults with atrial fibrillation. Cochrane database of systematic reviews (Online) 2024;9:CD011197. 10.1002/14651858.CD011197.pub3 PMC11406592 39287086

[19] Buckley BJR, Risom SS, Boidin M, Lip GYH, Thijssen DHJ. Atrial fibrillation specific exercise rehabilitation: are we there yet? J Pers Med 2022;12:610. 35455726 10.3390/jpm12040610 PMC9029299

[20] AbuElkhair A, Boidin M, Buckley BJR, Lane DA, Williams NH, Thijssen D, Lip GYH, Barraclough DL. Effects of different exercise types on quality of life for patients with atrial fibrillation: a systematic review and meta-analysis. J Cardiovasc Med (Hagerstown) 2023;24:87–95. 36583977 10.2459/JCM.0000000000001386

[21] Mohanty S, Mohanty P, Tamaki M, Natale V, Gianni C, Trivedi C, Gokoglan Y, Biase LDI, Natale A. Differential association of exercise intensity with risk of atrial fibrillation in men and women: evidence from a meta-analysis. J Cardiovasc Electrophysiol 2016;27:1021–1029. 27245609 10.1111/jce.13023

[22] Park CS, Choi EK, Kyung D, Yoo J, Ahn H-J, Kwon S, Lee S-R, Oh S, Lip GYH. Physical activity changes and the risk of incident atrial fibrillation in patients with type 2 diabetes mellitus: a nationwide longitudinal follow-up cohort study of 1.8 million subjects. Diabetes care 2023;46:434–440. 36469745 10.2337/dc22-1655

[23] Ekelund U, Steene-Johannessen J, Brown WJ, Fagerland MW, Owen N, Powell KE, Bauman A, Lee I-M; Lancet Physical Activity Series 2 Executive Committee; Lancet Sedentary Behaviour Working Group. Does physical activity attenuate, or even eliminate, the detrimental association of sitting time with mortality? A harmonised meta-analysis of data from more than 1 million men and women. Lancet 2016;388:1302–1310. 27475271 10.1016/S0140-6736(16)30370-1

[24] Kubota Y, Alonso A, Shah AM, Chen LY, Folsom AR. Television watching as sedentary behavior and atrial fibrillation: the atherosclerosis risk in communities study. J Phys Act Health 2018;15:895–899. 30463480 10.1123/jpah.2018-0064 PMC6351420

[25] Boursiquot BC, Bellettiere J, LaMonte MJ, LaCroix AZ, Perez MV. Sedentary behavior and atrial fibrillation in older women: the OPACH study. J Am Heart Assoc 2022;11:e023833. 35253465 10.1161/JAHA.121.023833 PMC9075327

[26] Eijsvogels TMH, Fernandez AB, Thompson PD. Are there deleterious cardiac effects of acute and chronic endurance exercise? Physiol Rev 2016;96:99–125. 26607287 10.1152/physrev.00029.2014 PMC4698394

[27] Buckley BJR, Lip GYH, Thijssen DHJ. The counterintuitive role of exercise in the prevention and cause of atrial fibrillation. Am J Physiol 2020;319:H1051–H1058. 10.1152/ajpheart.00509.2020 32946289

[28] Newman W, Parry-Williams G, Wiles J, Edwards J, Hulbert S, Kipourou K, Papadakis M, Sharma R, O'Driscoll J. Risk of atrial fibrillation in athletes: a systematic review and meta-analysis. Br J Sports Med 2021;55:1233–1238. 34253538 10.1136/bjsports-2021-103994

[29] Andrade JG, Deyell MW, Lee AYK, Macle L. Sex differences in atrial fibrillation. Can J Cardiol 2018;34:429–436. 29455950 10.1016/j.cjca.2017.11.022

[30] Morseth B, Graff-Iversen S, Jacobsen BK, Jørgensen L, Nyrnes A, Thelle DA, Vestergaard P, Løchen M-L. Physical activity, resting heart rate, and atrial fibrillation: the Tromso Study. Eur Heart J 2016;37:2307–2313. 26966149 10.1093/eurheartj/ehw059 PMC4986028

[31] Buckley BJR, Harrison SL, Fazio-Eynullayeva E, Underhill P, Lane DA, Thijssen DHJ, Lip GYH. Association of exercise-based cardiac rehabilitation with progression of paroxysmal to sustained atrial fibrillation. J Clin Med 2021;10:435. 33498648 10.3390/jcm10030435 PMC7865453

[32] Elliott AD, Verdicchio CV, Mahajan R, Middeldorp ME, Gallagher C, Mishima RS, Hendriks JML, Pathak RK, Thomas G, Lau DH, Sanders P. An exercise and physical activity program in patients with atrial fibrillation: the ACTIVE-AF randomized controlled trial. JACC Clin Electrophysiol 2023;9:455–465. 36752479 10.1016/j.jacep.2022.12.002

[33] Malmo V, Nes BM, Amundsen BH, Tjonna A-E, Stoylen A, Rossvoll O, Wisloff U, Loennechen JP. Aerobic interval training reduces the burden of atrial fibrillation in the short term: a randomized trial. Circulation 2016;133:466–473. 26733609 10.1161/CIRCULATIONAHA.115.018220

[34] Reed JL, Terada T, Vidal-Almela S, Tulloch HE, Mistura M, Birnie DH, Wells GA, Nair GM, Hans H, Way KL, Chirico D, O'Neill CD, Pipe AL. Effect of high-intensity interval training in patients with atrial fibrillation: a randomized clinical trial. JAMA Netw Open 2022;5:e2239380. 36315143 10.1001/jamanetworkopen.2022.39380 PMC9623436

[35] Luo N, Merrill P, Parikh KS, Whellan DJ, Piña IL, Fiuzat M, Kraus WE, Kitzman DW, Keteyian SJ, O'Connor CM, Mentz RJ. Exercise training in patients with chronic heart failure and atrial fibrillation. J Am Coll Cardiol 2017;69:1683–1691. 28359513 10.1016/j.jacc.2017.01.032 PMC5380238

[36] Pathak RK, Middeldorp ME, Lau DH, Mehta AB, Mahajan R, Twomey D, Alasady M, Hanley L, Antic NA, McEvoy RD, Kalman JM, Abhayaratna WP, Sanders P. Aggressive risk factor reduction study for atrial fibrillation and implications for the outcome of ablation: the ARREST-AF cohort study. J Am Coll Cardiol 2014;64:2222–2231. 25456757 10.1016/j.jacc.2014.09.028

[37] Pathak RK, Elliott A, Middeldorp ME, Meredith M, Mehta AB, Mahajan R, Hendriks JML, Twomey D, Kalman JM, Abhayaratna WP, Lau DH, Sanders P. Impact of CARDIOrespiratory FITness on arrhythmia recurrence in obese individuals with atrial fibrillation: the CARDIO-FIT study. J Am Coll Cardiol 2015;66:985–996. 26113406 10.1016/j.jacc.2015.06.488

[38] Qureshi WT, Alirhayim Z, Blaha MJ, Juraschek SP, Keteyian SJ, Brawner CA, Al-Mallah MH. Response to letter regarding article, Cardiorespiratory fitness and risk of incident atrial fibrillation: results from the Henry Ford exercise testing (FIT) project. Circulation 2015;131:1827–1834. 25904645 10.1161/CIRCULATIONAHA.114.014833

[39] Garnvik LE, Malmo V, Janszky I, Ellekjær H, Wisløff U, Loennechen JP, Nes BM. Physical activity, cardiorespiratory fitness, and cardiovascular outcomes in individuals with atrial fibrillation: the HUNT study. Eur Heart J 2020;41:1467–1475. 32047884 10.1093/eurheartj/ehaa032 PMC7320825

[40] Ahn H-J, Lee S-R, Choi E-K, Han K-D, Jung J-H, Lim J-H, Yun J-P, Kwon S, Oh S, Lip GYH. Association between exercise habits and stroke, heart failure, and mortality in Korean patients with incident atrial fibrillation: a nationwide population-based cohort study. PLoS Med 2021;18:e1003659. 34101730 10.1371/journal.pmed.1003659 PMC8219164

[41] Buckley BJR, Harrison SL, Fazio-Eynullayeva E, Underhill P, Lane DA, Thijssen DHJ, Lip GYH. Exercise-based cardiac rehabilitation and all-cause mortality among patients with atrial fibrillation. J Am Heart Assoc 2021;10:e020804. 34096332 10.1161/JAHA.121.020804 PMC8477861

[42] Buckley BJR, Lip GYH. Current concepts: comprehensive “cardiovascular health” rehabilitation—an integrated approach to improve secondary prevention and rehabilitation of cardiovascular diseases. Thromb Haemost 2022;122:1966–1968. 36307101 10.1055/s-0042-1757403

[43] Kraal JJ, Vromen T, Spee R, Kemps HMC, Peek N. The influence of training characteristics on the effect of exercise training in patients with coronary artery disease: systematic review and meta-regression analysis. Int J Cardiol 2017;245:52–58. 28735757 10.1016/j.ijcard.2017.07.051

[44] Franklin BA, Quindry J. High level physical activity in cardiac rehabilitation: implications for exercise training and leisure-time pursuits. Prog Cardiovasc Dis 2022;70:22–32. 34971650 10.1016/j.pcad.2021.12.005

[45] Leo DG, Buckley BJR, Chowdhury M, Harrison SL, Isanejad M, Lip GYH, Wright DJ, Lane DA; TAILOR investigators. Interactive remote patient monitoring devices for managing chronic health conditions: systematic review and meta-analysis. J Med Internet Res 2022;24:e35508. 36326818 10.2196/35508 PMC9673001

[46] McDonagh ST, Dalal H, Moore S, Clark CE, Dean SG, Jolly K, Cowie A, Afzal J, Taylor RS. Home-based versus centre-based cardiac rehabilitation. Cochrane database of systematic reviews (Online) 2023;10:CD007130. 10.1002/14651858.CD007130.pub5 PMC10604509 37888805

[47] Taylor RS, Dalal HM, Zwisler AD. Cardiac rehabilitation for heart failure: ‘Cinderella’ or evidence-based pillar of care? Eur Heart J 2023;44:1511–1518. 36905176 10.1093/eurheartj/ehad118 PMC10149531

[48] Gravely-Witte S, Leung YW, Nariani R, Tamim H, Oh P, Chan VM, Grace SL. Effects of cardiac rehabilitation referral strategies on referral and enrollment rates. Nat Rev Cardiol 2010;7:87–96. 19997077 10.1038/nrcardio.2009.223 PMC4474642

[49] Grace SL, Russell KL, Reid RD, Oh P, Anand S, Rush J, Williamson K, Gupta M, Alter DA, Stewart DE; Cardiac Rehabilitation Care Continuity Through Automatic Referral Evaluation (CRCARE) Investigators. Effect of cardiac rehabilitation referral strategies on utilization rates: a prospective, controlled study. Arch Intern Med 2011;171:235–241. 21325114 10.1001/archinternmed.2010.501

[50] Talha KM, Pandey A, Fudim M, Butler J, Anker SD, Khan MS. Frailty and heart failure: state-of-the-art review. J Cachexia Sarcopenia Muscle 2023;14:1959–1972. 37586848 10.1002/jcsm.13306 PMC10570089

[51] Purcell C, Dibben G, Boon MH, Matthews L, Palmer VJ, Thomson M, Smillie S, Simpson SA, Taylor RS. Social network interventions to support cardiac rehabilitation and secondary prevention in the management of people with heart disease. Cochrane database of systematic reviews (Online) 2023;6:CD013820. 10.1002/14651858.CD013820.pub2 PMC10305790 37378598

[52] Ragupathi L, Stribling J, Yakunina Y, Fuster V, McLaughlin MA, Vedanthan R. Availability, use, and barriers to cardiac rehabilitation in LMIC. Glob Heart 2017;12:323–334 e10. 28302548 10.1016/j.gheart.2016.09.004

[53] Mishima RS, Ariyaratnam JP, Pitman BM, Malik V, Emami M, McNamee O, Stokes MB, Lau DH, Sanders P, Elliott AD. Cardiorespiratory fitness, obesity and left atrial function in patients with atrial fibrillation. Int J Cardiol Heart Vasc 2022;42:101083. 35971520 10.1016/j.ijcha.2022.101083 PMC9375161

[54] Elliott AD, Ariyaratnam J, Howden EJ, La Gerche A, Sanders P. Influence of exercise training on the left atrium: implications for atrial fibrillation, heart failure, and stroke. American Journal of Physiology 2023;325:H822–H836. 37505470 10.1152/ajpheart.00322.2023

[55] Guasch E, Benito B, Qi X, Cifelli C, Naud P, Shi Y, Mighiu A, Tardif J-C, Tadevosyan A, Chen Y, Gillis M-A, Iwasaki Y-K, Dobrev D, Mont L, Heximer S, Nattel S. Atrial fibrillation promotion by endurance exercise: demonstration and mechanistic exploration in an animal model. J Am Coll Cardiol 2013;62:68–77. 23583240 10.1016/j.jacc.2013.01.091

[56] Abed HS, Wittert GA, Leong DP, Shirazi MG, Bahrami B, Middeldorp ME, Lorimer MF, Lau DH, Antic NA, Brooks AG, Abhayaratna WP, Kalman JM, Sanders P. Effect of weight reduction and cardiometabolic risk factor management on symptom burden and severity in patients with atrial fibrillation: a randomized clinical trial. JAMA 2013;310:2050–2060. 24240932 10.1001/jama.2013.280521

[57] Abidi AM, Mujaddadi A, Raza S, Moiz JA. Effect of physical exercise on cardiac autonomic modulation in hypertensive individuals: a systematic review and meta-analysis. Curr Hypertens Rev 2023;19:149–172. 37563821 10.2174/1573402119666230803090330

[58] Zhang Y, Ren P, Tang A, Dong L, Hu X, Wang H, Xu F. Efficacy of exercise rehabilitation in patients with atrial fibrillation after radiofrequency ablation: a meta-analysis of randomized controlled trials. Evid Based Complement Alternat Med 2022;2022:9714252. 36248413 10.1155/2022/9714252 PMC9568312

[59] Miguel-Dos-Santos R, Moreira JBN, Loennechen JP, Wisloff U, Mesquita T. Exercising immune cells: the immunomodulatory role of exercise on atrial fibrillation. Prog Cardiovasc Dis 2021;68:52–59. 34274371 10.1016/j.pcad.2021.07.008

[60] Kato M, Ogano M, Mori Y, Kochi K, Morimoto D, Kito K, Green FN, Tsukamoto T, Kubo A, Takagi H, Tanabe J. Exercise-based cardiac rehabilitation for patients with catheter ablation for persistent atrial fibrillation: a randomized controlled clinical trial. Eur J Prev Cardiol 2019;26:1931–1940. 31272205 10.1177/2047487319859974

[61] Shantsila E, Choi EK, Lane DA, Joung B, Lip GYH. Atrial fibrillation: comorbidities, lifestyle, and patient factors. Lancet Reg Health Eur 2024;37:100784. 38362547 10.1016/j.lanepe.2023.100784 PMC10866737

[62] Shaikh AY, Wang N, Yin X, Larson MG, Vasan RS, Hamburg NM, Magnani JW, Ellinor PT, Lubitz SA, Mitchell GF, Benjamin EJ, McManus DD. Relations of arterial stiffness and brachial flow-mediated dilation with new-onset atrial fibrillation: the Framingham Heart Study. Hypertension 2016;68:590–596. 27456517 10.1161/HYPERTENSIONAHA.116.07650

[63] Kim S, Lee S, Han D, Jeong I, Lee H-H, Koh Y, Chung SG, Kim K. One-year aerobic interval training improves endothelial dysfunction in patients with atrial fibrillation: a randomized trial. Intern Med 2023;62:2465–2474. 36631093 10.2169/internalmedicine.0947-22 PMC10518561

[64] Lin ML, Fu TC, Hsu CC, Huang SC, Lin YT, Wang JS. Cycling exercise training enhances platelet mitochondrial bioenergetics in patients with peripheral arterial disease: a randomized controlled trial. Thromb Haemost 2021;121:900–912. 33421964 10.1055/s-0040-1722191

[65] Van Gelder IC, Rienstra M, Bunting KV, Casado-Arroyo R, Caso V, Crijns HJGM, De Potter TJR, Dwight J, Guasti L, Hanke T, Jaarsma T, Lettino M, Løchen M-L, Lumbers RT, Maesen B, Mølgaard I, Rosano GMC, Sanders P, Schnabel RB, Suwalski P, Svennberg E, Tamargo J, Tica O, Traykov V, Tzeis S, Kotecha D; ESC Scientific Document Group. 2024 ESC Guidelines for the management of atrial fibrillation developed in collaboration with the European Association for Cardio-Thoracic Surgery (EACTS): developed by the task force for the management of atrial fibrillation of the European Society of Cardiology (ESC), with the special contribution of the European Heart Rhythm Association (EHRA) of the ESC. Endorsed by the European Stroke Organisation (ESO). Eur Heart J 2024;45:3314–3414. 39210723 10.1093/eurheartj/ehae176

